# Supplementary material for: Phosphodiesterase-5 inhibitors use and risk for mortality and metastases among male patients with colorectal cancer
Source: Nat Commun. 2020 Jun 24;11:3191. doi: 10.1038/s41467-020-17028-4 (PMC7314744; doi:10.1038/s41467-020-17028-4)
Supplement: Supplementary file 1 — Supplementary Information [file 41467_2020_17028_MOESM1_ESM.pdf]

SUPPLEMENTARY INFORMATION for Phosphodiesterase-5 inhibitors use and risk for mortality and metastasis among male patients with colorectal cancer by Huang, et al.

Supplementary Tables

Supplementary Table 1. Sensitivity analyses of hazard ratios (HRs) among male colorectal cancer patients

Supplementary Table 2. Stratified analyses by receiving open surgery of sensitivity analyses

Supplementary Figures

Supplementary Figure 1. Flowchart of patients in this respective cohort study

Supplementary Figure 2. Matched cohort design

Supplementary Table 1. Sensitivity analyses of hazard ratios (HRs) among male colorectal cancer patients

**Characteristics**	No. of patients	Person-years	Outcome	IR, 1000 person-years	Crude HR	95%CI	P value	Adjusted HR[a]	95%CI	P value
**Sensitivity 1** [b]										
**Death due to colorectal cancer**										
Without alprostadil	10525	50314	1729	34.36	1.00	-	-	1.00	-	-
Post-diagnostic use of alprostadil	68	265	9	33.92	1.06	0.55-2.04	0.863	1.02	0.53-1.97	0.955
**Metastasis**										
Without alprostadil	10525	48472	2101	43.34	1.00	-	-	1.00	-	-
Post-diagnostic use of alprostadil	68	243	20	82.28	1.62	1.01-2.57	0.043	1.35	0.85-2.15	0.21
**Sensitivity 2** [c]										
**Death due to colorectal cancer**										
Without PDE5 inhibitors	5455	31110	851	27.35	1.00	-	-	1.00	-	-
Post-diagnostic use of PDE5 inhibitors	1136	6908	116	16.79	0.69	0.56-0.84	<0.001	0.76	0.62-0.93	0.007
**Metastasis**										
Without PDE5 inhibitors	5455	29900	1307	43.71	1.00	-	-	1.00	-	-
Post-diagnostic use of PDE5 inhibitors	1136	6615	230	34.77	0.81	0.70-0.94	0.005	0.88	0.76-1.02	0.097
**Sensitivity 3** [d]										
**Death due to colorectal cancer**										
Without PDE5 inhibitors	11329	52563	1987	37.80	1.00	-	-	1.00	-	-
Post-diagnostic use of PDE5 inhibitors	1136	5743	116	20.20	0.71	0.59-0.85	<0.001	0.90	0.75-1.09	0.297
**Metastasis**										
Without PDE5 inhibitors	11329	49082	2523	51.40	1.00	-	-	1.00	-	-
Post-diagnostic use of PDE5 inhibitors	1136	5450	230	42.20	0.96	0.83-1.10	0.533	0.92	0.81-1.06	0.272
**Sensitivity 4** [e]										
**Death due to colorectal cancer**										
Without PDE5 inhibitors	7978	38953	1350	34.66	1.00	-	-	1.00	-	-
Post-diagnostic use of PDE5 inhibitors	922	4648	95	20.44	0.67	0.54-0.82	0.002	0.88	0.71-1.09	0.255

Continuous

**Metastasis**										
Without PDE5 inhibitors	7978	36318	1892	52.09	1.00	-	-	1.00	-	-
Post-diagnostic use of PDE5 inhibitors	922	4429	191	43.13	0.83	0.71-0.97	0.021	0.87	0.74-1.02	0.089
**Sensitivity 5** [f]										
**Death due to colorectal cancer**										
Without PDE5 inhibitors	16857	65475	5720	87.36	1.00	-	-	1.00	-	-
Post-diagnostic use of PDE5 inhibitors	1353	6704	197	29.39	0.48	0.42-0.56	<0.001	0.70	0.60-0.80	<0.001
**Metastasis**										
Without PDE5 inhibitors	16857	60271	6431	106.70	1.00	-	-	1.00	-	-
Post-diagnostic use of PDE5 inhibitors	1353	6297	353	56.06	0.67	0.60-0.75	<0.001	0.80	0.70-0.88	<0.001
**Sensitivity 6** [g]										
**Death due to colorectal cancer**										
Without PDE5 inhibitors	10312	50455	1597	31.65	1.00	-	-	1.00	-	-
Post-diagnostic use of PDE5 inhibitors	1091	5555	113	20.34	0.66	0.54-0.80	<0.001	0.82	0.68-1.00	0.052
**Metastasis**										
Without PDE5 inhibitors	10312	48890	2024	41.40	1.00	-	-	1.00	-	-
Post-diagnostic use of PDE5 inhibitors	1091	5457	199	36.47	0.80	0.69-0.93	0.003	0.81	0.69-0.94	0.006
**Sensitivity 7** [h]										
**Death due to colorectal cancer**										
Without PDE5 inhibitors	7723	38195	1096	28.69	1.00	-	-	1.00	-	-
Post-operative use of PDE5 inhibitors	156	719	11	15.29	0.60	0.33-1.09	0.098	0.80	0.44-1.45	0.461
**Metastasis**										
Without PDE5 inhibitors	7723	37482	1096	29.24	1.00	-	-	1.00	-	-
Post-operative use of PDE5 inhibitors	156	707	15	21.21	0.53	0.31-0.91	0.02	0.52	0.31-0.89	0.017

[a] Adjusted for age at diagnosis, year of diagnosis, cancer stage at diagnosis, birth country, marital status, highest education, income, prescription of other medicines (including aspirin, steroid and statin), depression and Charlson Comorbidity Index.

[b] Sensitivity analysis 1: Association between post-diagnostic use of alprostadil and prognosis among patients without using PDE5 inhibitors using time-dependent Cox regression.

[c] Sensitivity analysis 2: Association between post-diagnostic use of PDE5 inhibitors and prognosis in matched cohort design using Cox regression.

[d] Sensitivity analysis 3: Association between post-diagnostic use of PDE5 inhibitors and prognosis using time-dependent Cox regression with competing risk model.
[e] Sensitivity analysis 4: Association between post-diagnostic use of PDE5 inhibitors and prognosis among patients with Charlson Comorbidity Index = 0 using time-dependent Cox regression.
[f] Sensitivity analysis 5: Association between post-diagnostic use of PDE5 inhibitors and prognosis after including patients who were diagnosed with colorectal cancer at stage IV or unknown stage using time-dependent Cox regression.
[g] Sensitivity analysis 6: Association between post-diagnostic use of PDE5 inhibitors and prognosis after excluding patients with no more than six months of follow-up using time-dependent Cox regression.
[h] Sensitivity analysis 7: Association between solely post-operative use of PDE5 inhibitors and prognosis among patients with open surgery using time-dependent Cox regression.

Supplementary Table 2. Stratified analyses by receiving open surgery of sensitivity analyses

Characteristics	Crude HR	95%CI	P value	Adjusted HR [a]	95%CI	P value
**Matched cohort design**[b]						
Death due to CRC						
Without open surgery	0.87	0.65-1.18	0.383	0.91	0.67-1.24	0.548
Open surgery	0.61	0.47-0.79	<0.001	0.68	0.52-0.88	0.004
Metastasis						
Without open surgery	1.02	0.83-1.25	0.858	1.14	0.92-1.41	0.215
Open surgery	0.71	0.58-0.88	0.001	0.78	0.63-0.96	0.020
**Competing risk model**[c]						
Death due to CRC						
Without open surgery	0.84	0.63-1.11	0.222	1.14	0.85-1.51	0.383
Open surgery	0.64	0.50-0.81	<0.001	0.81	0.63-1.03	0.091
Metastasis						
Without open surgery	1.11	0.92-1.35	0.286	1.18	0.99-1.42	0.066
Open surgery	0.85	0.70-1.03	0.098	0.80	0.66-0.98	0.034
**CCI=0**[d]						
Death due to CRC						
Without open surgery	0.73	0.53-1.02	0.07	1.04	0.74-1.46	0.832
Open surgery	0.64	0.49-0.84	0.001	0.85	0.65-1.12	0.245
Metastasis						
Without open surgery	0.92	0.74-1.15	0.469	1.10	0.87-1.37	0.429
Open surgery	0.77	0.62-0.96	0.021	0.80	0.64-0.99	0.045

[a] Adjusted for age at diagnosis, year of diagnosis, cancer stage at diagnosis, birth country, marital status, highest education, income, prescription of other medicines (including aspirin, steroid and statin), depression and Charlson Comorbidity Index.
[b] Cox regression was used to calculate HRs and 95% CIs. [c] Time-dependent Cox regression with competing risk model was used to calculate HRs and 95% CIs. [d] Time-dependent Cox regression was used to calculate HRs and 95% CIs.

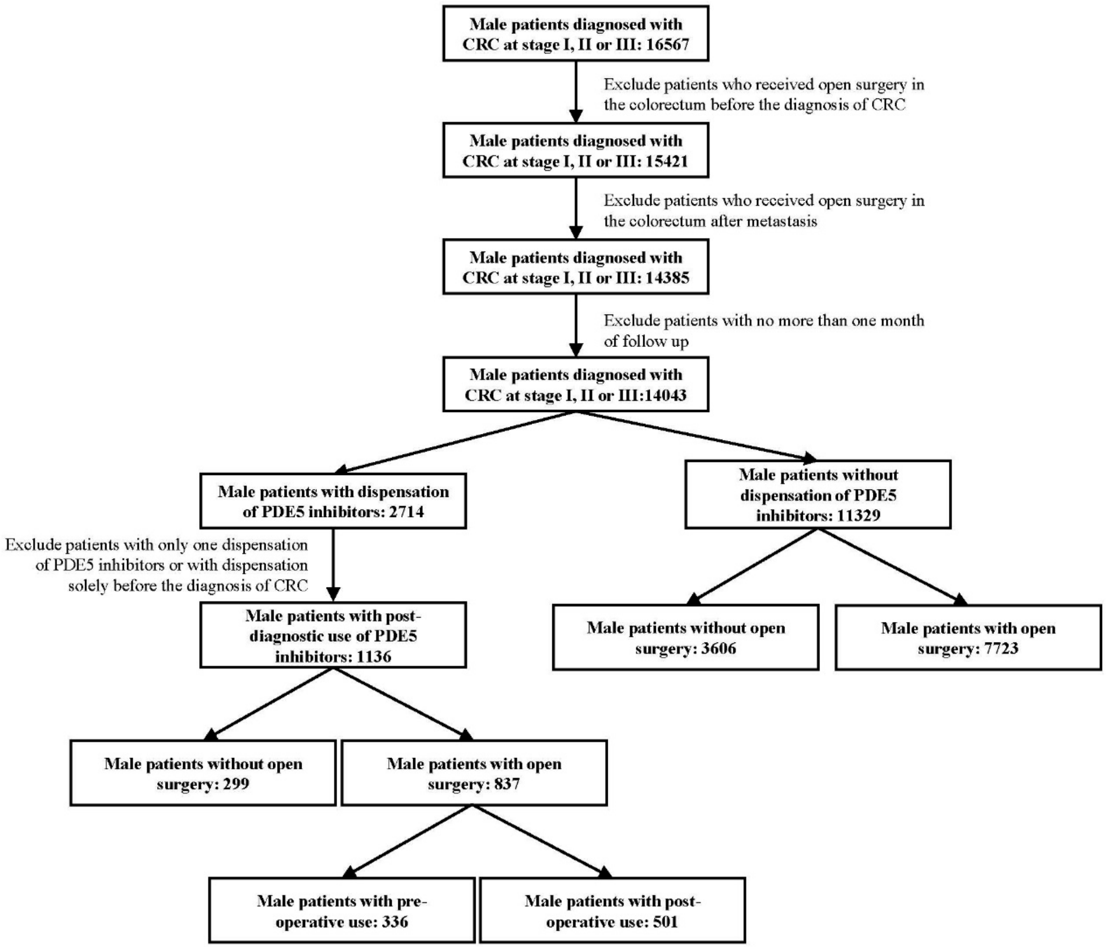

Supplementary Figure 1. Flowchart of patients in this respective cohort study

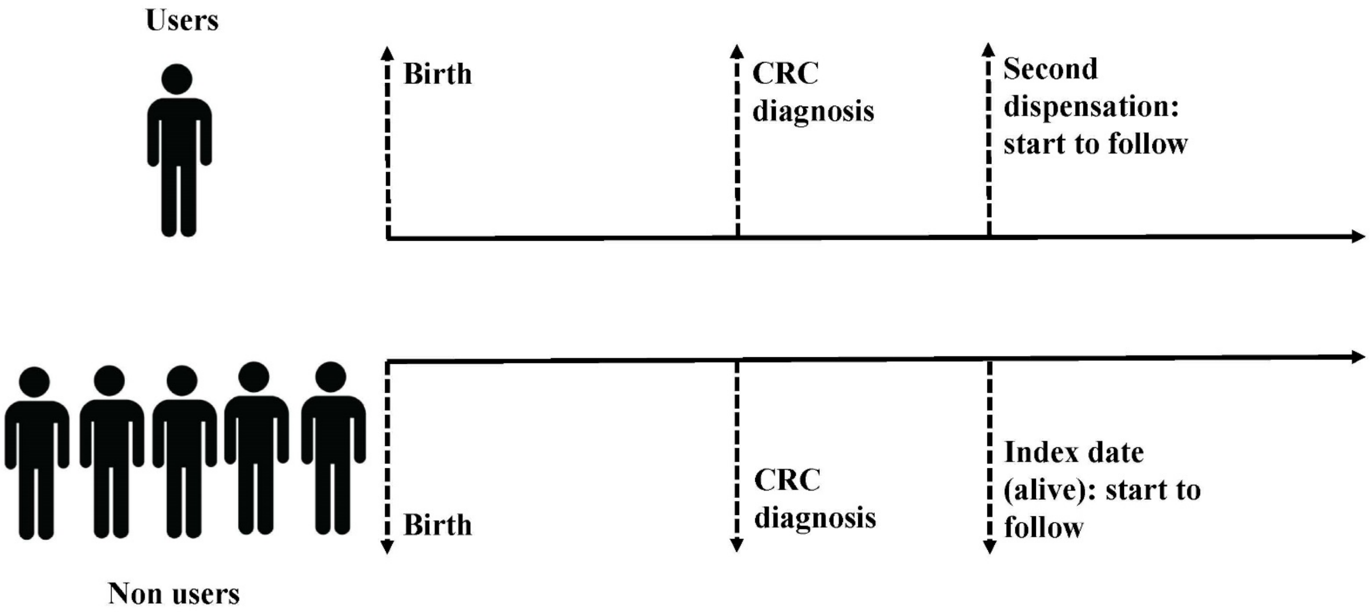

Supplementary Figure 2. Mathched cohort design
