# Supplementary material for: Phosphodiesterase-5 inhibitors use and risk for mortality and metastases among male patients with colorectal cancer
Source: Nat Commun. 2020 Jun 24;11:3191. doi: 10.1038/s41467-020-17028-4 (PMC7314744; doi:10.1038/s41467-020-17028-4)
Supplement: Supplementary file 3 — Reporting Summary [file 41467_2020_17028_MOESM3_ESM.pdf]

nature research

Corresponding author(s): Wuqing Huang & Jianguang Ji

Last updated by author(s): May 21, 2020

Reporting Summary

Nature Research wishes to improve the reproducibility of the work that we publish. This form provides structure for consistency and transparency in reporting. For further information on Nature Research policies, see our Editorial Policies and the Editorial Policy Checklist.

Statistics

For all statistical analyses, confirm that the following items are present in the figure legend, table legend, main text, or Methods section.

n/a	Confirmed	
☐	☒	The exact sample size (n) for each experimental group/condition, given as a discrete number and unit of measurement
☐	☒	A statement on whether measurements were taken from distinct samples or whether the same sample was measured repeatedly
☐	☒	The statistical test(s) used AND whether they are one- or two-sided<br>*Only common tests should be described solely by name; describe more complex techniques in the Methods section.*
☐	☒	A description of all covariates tested
☐	☒	A description of any assumptions or corrections, such as tests of normality and adjustment for multiple comparisons
☐	☒	A full description of the statistical parameters including central tendency (e.g. means) or other basic estimates (e.g. regression coefficient) AND variation (e.g. standard deviation) or associated estimates of uncertainty (e.g. confidence intervals)
☐	☒	For null hypothesis testing, the test statistic (e.g. F, t, r) with confidence intervals, effect sizes, degrees of freedom and P value noted<br>*Give P values as exact values whenever suitable.*
☒	☐	For Bayesian analysis, information on the choice of priors and Markov chain Monte Carlo settings
☒	☐	For hierarchical and complex designs, identification of the appropriate level for tests and full reporting of outcomes
☐	☒	Estimates of effect sizes (e.g. Cohen's d, Pearson's r), indicating how they were calculated

Our web collection on statistics for biologists contains articles on many of the points above.

Software and code

Policy information about availability of computer code

Data collection	SAS version 9.4 was used to link registers used in this study. Data of gene expression were retrieved from http://xena.ucsc.edu/.
Data analysis	Statistical analyses were conducted using SAS version 9.4 and R version 3.6.0

For manuscripts utilizing custom algorithms or software that are central to the research but not yet described in published literature, software must be made available to editors and reviewers. We strongly encourage code deposition in a community repository (e.g. GitHub). See the Nature Research guidelines for submitting code & software for further information.

Data

Policy information about availability of data

All manuscripts must include a data availability statement. This statement should provide the following information, where applicable:

- Accession codes, unique identifiers, or web links for publicly available datasets
- A list of figures that have associated raw data
- A description of any restrictions on data availability

The data based on Swedish register are not publicly available due to Swedish law and protecting patients privacy, the combined set of data used for the analysis presented in this study can only be made available from the appropriate Swedish authorities (the Swedish National Board of Health and Welfare (https://www.socialstyrelsen.se/en) and Statistics Sweden (https://www.scb.se/en), for researchers who meet the criteria for access to confidential. TCGA Colon and Rectal Cancer is combined from TCGA colon adenocarcinoma and rectum adenocarcinoma datasets, which can be accessed on UCSC Xena platform (https://tcga.xenahubs.net).

Field-specific reporting

Please select the one below that is the best fit for your research. If you are not sure, read the appropriate sections before making your selection.

☐ Life sciences ☒ Behavioural & social sciences ☐ Ecological, evolutionary & environmental sciences

For a reference copy of the document with all sections, see nature.com/documents/nr-reporting-summary-flat.pdf

Behavioural & social sciences study design

All studies must disclose on these points even when the disclosure is negative.

Study description	Quantitative retrospective cohort study based on Swedish registers
Research sample	All male patients diagnosed with CRC at stage I, II or III in Sweden between January 2005 and March 2014 were identified from Swedish Cancer Registry, this Register is estimated to cover 90% newly-diagnosed cancer cases in Sweden. A total of 12 465 male patients were included with the median age of 71.
Sampling strategy	We included all eligible male patients identified from Swedish Cancer Registry in the study, the registry have more than 90% coverage of the entire Swedish population.
Data collection	Data in Swedish Cancer Registry is collected through reports from clinicians, pathologists and cytologists in Sweden. It is compulsory for clinicians, pathologists and cytologists to report all newly diagnosed cancers to the Swedish Cancer Registry. The information of death from the Cause of Death Register is collected via a death certificate and a cause of death certificate which must be completed by a doctor and then sent to the National Board of Health and Welfare. Data from National Patient Register is regulated by the Health Data Register and related regulations It is compulsory for those who practice health care to submit information to National Patient Register. All data in the Swedish Prescribed Drug Register comes from the E-health authority. All trades that sell medicines are obliged to report sales data to the E-health authority. In addition, pharmacies must submit additional information to the E-Health Authority when a prescribed drug is dispensed. The E-health authority in turn submits information on prescription dispensations to the National Board of Health and Welfare.
Timing	Swedish Cancer Registry was created in 1958 and last updated in 2016. Cause of Death Register was created in 1961 and last updated in 2016. National Patient Register was developed in 1964 and last updated in 2016. Swedish Prescribed Drug Register was created in 2005 and last updated in 2014. Male patients diagnosed with CRC between January 2005 and March 2014 were included. Patients were followed up starting at the date of diagnosis with CRC, and ending at the time of occurrence of outcomes (death due to CRC or metastasis) or at the end of the follow-up period (December 2015), whichever came first.
Data exclusions	Patients were excluded if: (1) Patients who received open surgery in the colorectum before the diagnosis of CRC (n=1146) to minimize the impact from previous open surgery before CRC diagnosis; (2) Patients who received open surgery in the colorectum after metastasis (n=1036) to keep consistence in terms of the population when investigate the risk of death and metastasis; (3) Patients who were followed up no more than one month(n=342) to ensure enough follow-up for each patient; (4) Patients who only used PDES inhibitors before the diagnosis of CRC or patients with only one dispensation of PDE5 inhibitors (n=1578) to focus on exploring the effect of post-diagnostic use and take into consideration the possibility of non-adherence.
Non-participation	N/A, because datasets used in this study provides the basis for the official statistics, it is compulsory for relevant staff to report information.
Randomization	N/A, this is a observational cohort study, we divided population based on exposure at baseline.

Reporting for specific materials, systems and methods

We require information from authors about some types of materials, experimental systems and methods used in many studies. Here, indicate whether each material, system or method listed is relevant to your study. If you are not sure if a list item applies to your research, read the appropriate section before selecting a response.

Materials & experimental systems

n/a	Involved in the study	
☒	☐	Antibodies
☒	☐	Eukaryotic cell lines
☒	☐	Palaeontology and archaeology
☒	☐	Animals and other organisms
☐	☒	Human research participants
☒	☐	Clinical data
☒	☐	Dual use research of concern

Methods

n/a	Involved in the study	
☒	☐	ChIP-seq
☒	☐	Flow cytometry
☒	☐	MRI-based neuroimaging

Human research participants

Policy information about studies involving human research participants

Population characteristics	See above.
Recruitment	Male patients diagnosed with CRC were identified from Swedish Cancer Registry, which has an estimated over 90% coverage of the entire Swedish population. Female patients were not included as few females were prescribed with PDE5 inhibitors, which may affect the generalization of the results.
Ethics oversight	The Ethics Committee at Lund University approved (February 6, 2013) this nationwide cohort study (Dnr 2012/795). Written informed consent is not needed in Sweden for the register-based study.

Note that full information on the approval of the study protocol must also be provided in the manuscript.
